# Nurses experiences of adopting evidence-based practice in Saudi healthcare: organizational, educational, and cultural influences from qualitative focus groups

**DOI:** 10.1186/s12912-026-04768-9

**Published:** 2026-05-19

**Authors:** Amira Suliman AlAnizi, Seham Mansour Alyousef, Sami Abdulrahman Alhamidi

**Affiliations:** 1 Philosophy in Nursing Program Nursing College, King Saud University, Riyadh, Saudi Arabia; 2 Department of Community and Mental Health Nursing, Nursing College, King Saud University, Riyadh, Saudi Arabia; 3 Department of Maternal and Child Health Nursing, Nursing College, King Saud University, Riyadh, Saudi Arabia

**Keywords:** Evidence-based practice, Nursing, Johns Hopkins PET model, Framework-guided thematic analysis, Barriers, Facilitators, Knowledge translation, Organizational culture, Saudi Arabia

## Abstract

**Background:**

Evidence-based practice (EBP) is expected in nursing, and its implementation is still limited by workload, evidence access, and organizational culture. In Saudi settings, nurses report mixed readiness for EBP and recurring constraints related to resources, autonomy, and change processes in daily care. This exploratory study aimed to examine the perspectives of postgraduate nursing students (Master’s and doctoral level) on barriers and facilitators to EBP in the Saudi healthcare context, using the Johns Hopkins Practice Evidence Translation (PET) model as the analytic framework.

**Methods:**

A framework-guided thematic analysis of recorded focus group transcripts used deductive coding to PET domains (Practice, Evidence, Translation) with dual coding across two purposively sampled focus groups: Master’s-level nursing students (FG1; *n* = 5) and doctoral-level nursing students (FG2; *n* = 5), yielding *N* = 10 participants.

**Results:**

In Practice participants framed EBP as “not personal preference,” integrating research evidence, clinical experience, and patient preferences, and described adapting tools and protocols locally. Key practice barriers include bedside resistance when EBP steps were viewed as extra work and hierarchical norms that constrained nursing autonomy. In Evidence, participants reported variable EBP education and research literacy and inconsistent organizational access to journals and databases. In Translation, facilitators included leadership support and capacity-building, while barriers included multi-level resistance and slow approval processes.

**Conclusion:**

PET mapping highlights priorities to protect time for EBP work, build research skills, ensure reliable evidence access, and streamline translation pathways.

## Introduction

Evidence-based practice (EBP) is a core expectation for nursing because it supports safer, higher quality care by comparing clinical decisions with the best available research evidence, clinician expertise, and patient outcomes. The know-do gap is persistent: even when nurses value EBP, daily implementation is constrained by clinical workload and limited access to evidence resources. In Saudi Arabia context, scholars reported that nurses have moderate to high awareness of EBP principles, however, they implement is very low. This could be due to barriers in workload feasibility, limited research training, and restricted access [1–5]. Accordingly, this might situate Saudi nursing similar to the international know-do gap. This is usually shaped by both individual competency, organizational culture, resource provision, and leadership climate [6, 7]. In line with this understanding, Studies in settings describe barriers which include time pressure, competing demands, and limited institutional support, with facilitators, leadership engagement, training, and practical infrastructure for access and implementation [1–3].

Within Saudi Arabia, nursing and nursing administrator samples have reported mixed readiness for EBP, with common constraints related to workload feasibility, availability of resources, organizational processes, and variability in research preparation [1–3]. Organizational culture mainly how teams communicate, learn, and authorize practice change, can enable or suppress EBP behaviors [4–6]. These patterns align with broader evidence that leadership and culture shape nurses’ engagement with evidence to initiate or participate in change [7–12].

Despite this, important gaps remain to be present. First, Saudi studies commonly use quantitative survey designs to measure EBP beliefs and implementation rates. However, this might limit the insight into how nurses and nurse educators actually experience EBP in their daily work [1–3, 5]. Second, it could be argued that studies rarely examine the perspectives of nurses at different educational levels separately or comparatively. Although educational level has been identified as a significant predictor of EBP competency [4, 13]. Third, despite the importance and recognition of the Johns Hopkins Practice–Evidence–Translation (PET) model as an analytic framework for qualitative exploration of EBP in the Saudi context [3, 14], yet it was never used in studies that explore this topic.

Implementation supports also matter: educational interventions can improve EBP competence, but effects depend on reinforcement and workplace opportunities to apply skills [15]. Practical strategies, mentorship and structured training strengthen evidence skills and confidence [16]. Journal clubs and evidence forums are used as low cost implementation supports, although their value depends on participation, leadership endorsement, and relevance to local problems [17, 18]. Tools that measure EBP competencies can help diagnose needs and guide training plans [19].

To examine EBP barriers and facilitators in a way that is close to clinical work, frameworks that connect bedside practice to evidence and organizational translation are useful [14]. To address these gaps, a qualitative approach is needed in the search of capturing nurses lived experiences of EBP implementation. Furthermore, to illuminate contextual and cultural factors specific to Saudi healthcare. In the same vein, it might generate findings grounded in participant accounts rather than pre-defined survey scales [14]. Our study used the Johns Hopkins Practice–Evidence–Translation (PET) model to explore how nurses and nurse educators describe EBP implementation and what conditions enable or impede it in their settings [3]. Two focus groups were conducted: Master’s-level nursing students (FG1; *n* = 5) and doctoral-level nursing students (FG2; *n* = 5), enabling comparison across educational levels. This exploratory methos provides qualitative, PET-framework-guided on EBP experiences among postgraduate nursing students at King Saud University. This perspective is to complements the existing quantitative evidence base. In addition, this design help to identify research gaps in Saudi nursing [3].

## Methods

Purposive sampling was implemented to recruit the sample. Special concern was given to direct experience of EBP implementation in Saudi healthcare settings. Eligibility criteria include registered nurses or nursing faculty currently employed in a clinical or academic institution in Saudi Arabia, with at least 1 year of experience. Recruitment itself was conducted through professional networks and institutional contacts. Both focus groups were conducted at King Saud University, Riyadh, Saudi Arabia 2025. Participants in focus group1 were Master’s-level nursing students enrolled in the nursing program at King Saud University (*n* = 5). Participants in focus group 2 were doctoral-level nursing students at the same institution (*n* = 5). While all participants were enrolled at King Saud University, they have diverse clinical and academic backgrounds. Their work experience ranged from tertiary hospitals, primary healthcare centres, and university settings across Saudi Arabia (Table 1).

**Table 1:** Participant characteristics **(***N***=10 across two focus groups)**

Participant Code	Role	Setting / Institution	Years Exp.	Sector	EBP Training	Language
Focus Group 1 (FG1): Master’s-level Nursing Students — King Saud University, Riyadh, 2025 (*n* = 5)						
FG1-A	Clinical Nurse	King Saud University Medical Centre (KSUMC)	5 years	Government	Yes (workplace)	English Arabic
FG1-B	Clinical Nurse	National Guard Health Affairs — Paediatric Oncology Infusion Unit	5 years	Government	Not stated	English Arabic
FG1-C	Clinical Nurse	Ministry of Health (multi-hospital experience, 4 hospitals)	15 years	Government	Yes (workplace)	Arabic English
FG1-D	Clinical Nurse	King Saud University Medical Centre (KSUMC) — Quality Dept. liaison	Not stated	Government	Not stated	Arabic English
FG1-E	Clinical Nurse	King Saud University Medical Centre (KSUMC)	Not stated	Government	None	Arabic
**Focus Group 2 (FG2): Doctoral-level Nursing Students — King Saud University**,** Riyadh**,** 2025 (n** **= 5)**						
FG2-A	Clinical Instructor	Taibah University — College of Nursing (field training & clinical skills)	~ 4 years	Educational	Not stated	English Arabic
FG2-B	Primary Nurse (prev. Head Nurse / Quality / Education Nurse)	Second Health Cluster, Governmental Health Centre — King Faisal	12 years	Government	Not stated	English Arabic
FG2-C	Lecturer	Imam Abdulrahman bin Faisal University — Community & Mental Health Nursing	6 yrs academic + 3 yrs clinical	Educational	Not stated	Arabic
FG2-D	Clinical Nurse — Paediatric ICU / BACU	King Faisal Specialist Hospital, Jeddah Branch	Not stated	Government	Yes (mandatory annual module + workshop)	English
FG2-E	Nurse Educator / Faculty	Nursing College — Primary Healthcare clinics (7 clinics incl. ER, vaccination, well-baby, maternity)	16 years	Educational	Yes (studied in UK — Bachelor’s & Master’s)	Arabic/English

**Notes**: Participant names were not recorded in the transcript to protect confidentiality; codes FG1-A through FG1-E and FG2-A through FG2-E are assigned based on order of contribution. Years of experience marked ‘Not stated’ were not explicitly mentioned during the focus group introduction. All focus groups were conducted online in 2025 at King Saud University. EBP = Evidence-Based Practice. Sectors: Government = Ministry of Health / governmental hospitals; Educational = university / nursing college

A framework guided thematic analysis was conducted on the focus group transcript using the Johns Hopkins PET (Practice–Evidence–Translation) model as the a priori analytic framework. Both focus groups were moderated using the same structured questioning route. Data saturation is addressed via cross-group convergence statement in Discussion and Limitations. Also, voluntary participation and withdrawal rights were stated. To protect confidentiality, all participant identifiers in analytic outputs were replaced with letters (A, B, C …), and only role details stated in the transcript were retained for participant mapping; no demographic characteristics were inferred when not present in the dataset.

The unit of analysis was a meaning unit a phrase, sentence, or short paragraph expressing one coherent idea relevant to EBP implementation. Both transcripts were read repeatedly to achieve familiarization. Each segment was assigned to one PET domain; when a segment clearly contained more than one PET construct, dual coding in domains was applied to preserve meaning rather than forcing a single category placement. Codes were kept close to participants language and refined only when recurring PET relevant content was not represented by existing codes.

A structured codebook was developed iteratively within each PET domain, documenting for every code: a label, definition, inclusion and exclusion criteria, and an excerpt anchor from the transcript to ensure traceability from raw data to interpretation. Codes were then clustered within each PET domain into candidate themes based on semantic similarity and coherence. Each PET-domain theme was classified as a barrier, facilitator, or mixed condition for EBP based on how participants described its influence in practice. The analytic outputs include a participant mapping table, a PET theme barrier and facilitator matrix, and three PET-specific codebook tables, with a PET-structured results narrative that referenced these tables and remained grounded in the transcript data from both focus groups.

To ensure trustworthiness, credibility was supported by engagement with both transcripts through repeated reading and iterative codebook refinement. Dependability was addressed by maintaining a detailed audit trail of coding decisions. Confirmability was supported by grounding all interpretations in direct transcript excerpts with anchor quotes for each code. Transferability is supported by thick description of the study context, participant roles, and analytic procedures. Then, coders (A.A. and S.A.) coded dually: A.A. did the primary coding of all data, while S.A. did 20% of the coding (randomly selected meaning units from each focus group). Discrepancies were discussed and resolved iteratively until full agreement by both. Analysis was guided by the Johns Hopkins PET model (Dang et al., 2022) and the framework-guided thematic analysis approach (Ritchie and Spencer, 1994). Coding was primarily deductive, structured by PET domains; however, within-domain codes were allowed to emerge inductively and refined iteratively. Analysis was conducted manually. To assess data saturation, thematic convergence was monitored across both groups. So, saturation was achieved when FG2 (doctoral-level, *n* = 5) did not add new themes beyond those identified in FG1 (Master’s-level, *n* = 5). In addition, when additional coding of FG2 produced no new codes beyond those already represented in the codebook.

Reflexivity was addressed by acknowledging the first author’s (A.A.) positionality as a doctoral nursing student and faculty member who had no prior supervisory, evaluative, or collegial relationship with any participant. This outsider position to participants reduced the risk of social desirability bias and supported open disclosure during both focus group sessions.

## Results

Using a deductive, framework guided approach in line with the Johns Hopkins PET (Practice, Evidence, Translation) model, interviews were organized into PET domains to explain how evidence-based practice (EBP) was understood, and constrained in participants’ settings. Participants are anonymized by focus group and letter: Master’s-level group participants as FG1-A through FG1-E (*n* = 5), and doctoral-level group participants as FG2-A through FG2-E (*n* = 5), for a total of *N* = 10 participants.

### Direct participant quotations – Practice domain (from transcripts FG1 and FG2)

#### Theme PRA-1: EBP as not personal preference / evidence-guided decisions

**FG2-A (FG2, doctoral group):** *“Our practice as nurses is not based on favoritism nor is it based on any personal thing – it is based on evidence either through research or science.”*

**FG2-B (FG2, doctoral group): ***“Practice should not depend on my personal opinion or preference. Practice relies on our experience and also on updated research*,* meaning what science has reached. It should not be solely based on what we think is best for the patient – it depends on patient preference*,* our experience*,* and updated research.”*

**FG1-A (FG1, Master’s group):** *“Nursing supervision becomes based on awareness*,* not habit – meaning I do not implement any procedure just because we always do it this way.”*

#### Theme PRA-2: Evidence ‘experience’ patient *integration*

**FG2-B (FG2, doctoral group):** *“Practice relies on our experience and also on updated research, meaning what science has reached. It should not be solely based on what we think is best for the patient – it depends on patient preference, our experience, and updated research.”*

**FG2-A (FG2, doctoral group):** *“It is based on evidence either through research or science.”*

**FG1-B (FG1, Master’s group):** *“Nursing supervision becomes based on awareness, not habit – meaning do I not implement any procedure just because we always do it this way.”*

#### Theme PRA-3: Local adaptation of tools and protocols

**FG2-C (FG2, doctoral group):** *“We noticed that unplanned admissions to BACU increased. So we looked at the evidence to see how we could improve. We searched the literature and found that an early warning sign tool for the paediatric patient would allow early recognition of any patient deteriorating on the floor. We adapted the tool and implemented it across all paediatric areas and found a huge significant improvement in ambulatory admissions to the ICU.”*

**FG2-C (FG2, doctoral group):** *“We adapted a sedation scale called COMFORT – after a literature review we found it was the best option for our patients. We assessed sedation level before extubation*,* and so far over two years we have had zero unplanned extubations.”*

**FG1-B (FG1, Master’s group):** *“In the Paediatric Oncology Infusion Unit*,* we use Alteplase in case of a blocked central line. We read the latest literature and found that the dosing had completely changed – they increased the dose and the clot dissolved continuously from the first time. Its impact was huge*,* reducing visits to the ER and to the unit. So we revised the whole guideline based on evidence-based practice.”*

#### Theme PRA-4: Workload resistance at bedside

**FG2-C (FG2, doctoral group):** *“The floor nurses were against it – why do we have to do all these things? It is easier for us to call the RRT and have them come and assess. Why do we have to do it?”*

**FG1-C (FG1, Master’s group):** *“I expect that the staff themselves are not always ready. If you give them any new evidence or practice*,* it is difficult for them to let go of the previous one.”*

#### Theme PRA-5: Hierarchical practice culture limiting nursing autonomy

**FG2-D (FG2, doctoral group):** *“We have a cultural issue where hierarchy in hospitals means that in most environments*,* nurses are expected to simply take instructions from the doctor*,* without initiative or independence in decision-making. We are used to them just following orders. Some hospitals do not encourage nurses to research and discuss – the approach is simply ‘take orders and follow them’.”*

**FG2-E (FG2, doctoral group):** *“When the staff comes*,* he already has the knowledge or is updated. But when he discusses it with a senior*,* they are refused. As we say*,* ‘This is what we found from our fathers’ – we must not question it.”*

Practice: Participants framed EBP as clinical work that should not be driven by personal preference; instead, decisions were described as balancing updated research evidence, clinical experience, and patient preference (Table 2). This conceptualization positioned EBP as a practical decision-making method rather than an abstract research activity. Practice enactment was illustrated through examples of bedside, where participants described reviewing evidence and adapting tools for local use (Table 2). One account described adopting an early warning tool after observing increased unplanned admissions and reporting practice improvement following implementation. Another account described implementing a pediatric sedation assessment approach and a securement device after unplanned self-extubation events, with sustained reduction in adverse events in that unit context.

**Table 2:** Codebook PET domain practice

Code	Definition	Include	Exclude
PRA-1 Non-personal practice	EBP framed as not based on favoritism preference	Statements contrasting evidence vs. opinion	Training logistics without practice meaning
PRA-2 Evidence–experience patient integration	Practice decisions integrating research, experience, patient preference	Mentions of experience + patient preference	policy delays
PRA-3 Practice improvement via tools	Concrete practice change using tools, scales and protocols	Early warning tool; sedation assessment	Generic EBP is important
PRA-4 Workload feasibility barrier	Resistance due to extra steps burden	why we have to do all these things	Hierarchy-only barriers
PRA-5 Hierarchical practice culture	Reduced autonomy due to hierarchical norms	take instruction, without initiative; role acceptance	Database access issues

Practice-level barriers were prominent, as participants described bedside resistance when evidence-informed steps were experienced as additional workload or unnecessary tasks (Table 2). A hierarchical culture was described (limiting autonomy; follow instructions rather than initiate evidence-informed changes, and challenges in role acceptance) (Table 2). In summary, the Practice domain reveals a central tension. Both groups demonstrated genuine commitment evidenced by informed decisions and local adaptation. However, systemic barriers rooted in workload pressures and hierarchical norms are present. This tension was consistent across FG1 and FG2, suggesting it reflects structural features of Saudi healthcare.

### Direct participant quotations – Evidence domain (from transcripts FG1 and FG2)

#### Theme EVI-1: Evidence as updated research and science

**FG2-B (FG2, doctoral group):** *“Practice relies on our experience and also on updated research*,* meaning what science has reached. We follow it.”*

**FG2-D (FG2, doctoral group):** *“As a nurse*,* the priority comes from the evidence. When I run in the morning at work*,* I have to set priorities for patient care – and those priorities come from the evidence-based output.”*

#### Theme EVI-2: Research literacy gap

**FG2-D (FG2, doctoral group):** *“Many practitioners want to apply evidence*,* but unfortunately they lack basic research skills to determine if the evidence is appropriate or not.”*

**FG2-D (FG2, doctoral group):** *“When you go to the hospital*,* you find that some nurses lack the skills – how do they use it? How do they search for it? It is essential for employers to train practitioners in scientific research methodologies.”*

#### Theme EVI-3: Formal EBP education variability

**FG2-E (FG2, doctoral group): ***“Yes*,* during my Master’s studies*,* there was a full course on evidence-based practice.”*

**FG1-D (FG1, Master’s group):** *“Official training on evidence-based practice – I personally have never attended. I don’t know if the clinical resource nurses can provide it; I have never heard that there is a workshop on it.”*

#### Theme EVI-4: Access to evidence resources as structural constraint

**FG2-E (FG2, doctoral group):** *“If employers create a digital library for nursing staff*,* it would be a huge advantage. When you are a student at a university*,* you have access to the Saudi Digital Library. But in the workplace*,* these things are not available. When my account is suspended*,* or my account belongs to the university*,* I cannot search anymore – I cannot find free articles*,* I cannot find all the evidence I need. It is tiring and exhausting.”*

**FG1-E (FG1, Master’s group):** *“The natural keys – resources – are an effort for investigation and are available from the hospital*,* like the library at the university. But so far*,* we are using technology like computers*,* and we have hard copies that will be entered into the database.”*

Evidence: Evidence was commonly characterized as updated research and what science has reached, show currency and scientific grounding (Table 3). Participants also indicate variability in EBP preparation as some accounts referenced receiving formal EBP education, while others stressed that many practitioners want to apply EBP but lack basic research literacy, including how to evaluate whether evidence is appropriate. Access to evidence resources was raised as a structural constraint; one account recommended organizational provision of a digital library and described loss of database (Table 3). In summary, the Evidence domain highlights two interdependent constraints: First, a research literacy gap (more pronounced in FG1 Master’s-level participants who reported no formal EBP training). Second, a structural access gap affecting both groups. Together, these create a double barrier where nurses may lack both the competency to appraise evidence and the infrastructure to retrieve it. This combination can undermines EBP implementation regardless of motivation.

**Table 3:** Codebook PET domain evidence

Code	Definition	Include	Exclude
EVI-1 Evidence as updated science	Evidence described as updated research	updated research; what science has reached	Pure implementation steps
EVI-2 Research literacy gap	Difficulty searching suitability of evidence	lack basic research skill, determine if evidence is appropriate	Policy delays not tied to skills
EVI-3 Formal education variability	Formal EBP training reported by some	EBP course in graduate study	Bedside tool use without education mention
EVI-4 Evidence access constraint	Limited access to journals/databases; need digital library	Digital library suggestion; access suspended	Culture/hierarchy issues
EVI-5 Evidence-informed patient education	Using evidence language to guide patient advice	information should be evidence-based latest research	Non-evidence patient counseling

### Direct participant quotations – Translation domain (from transcripts FG1 and FG2)

#### Theme TRN-1: Organisational EBP infrastructure and capacity-building

**FG2-C (FG2, doctoral group):** *“We have a mandatory module for evidence-based practice – we have to do it every year. It is not optional*,* it is mandatory. During recontracting*,* they will ask you for the certificate. Additionally*,* we run an evidence-based practice workshop*,* and any newly graduated staff must complete their own evidence-based project during their first year of employment.”*

**FG1-F (FG1, Master’s group):** *“In KSUMC*,* there are available courses conducted regularly for employees at various levels. After you take the course on how to start or create evidence-based practice*,* along with having scientific data*,* they will publish it as evidence-based practice.”*

#### Theme TRN-2: Leadership prioritisation and annual goals

**FG2-C (FG2, doctoral group):** *“One of our strategic plans is research and evidence-based practice. Each unit sets its own annual goal: to present or complete one evidence-based project and to participate in a journal club. At the end of the year*,* they participate in a competition – nursing leaders are recognised for which unit completed the most evidence-based projects.”*

**FG1-G (FG1, Master’s group):** *“Encouragement is present but I consider it somewhat moderate – the shortage reduces the time available for practical application or attending training courses that help nurses use evidence in their daily decisions.”*

#### Theme TRN-4 & TRN-5: Multi-level resistance and governance delay

**FG2-C (FG2, doctoral group):** *“Once we found a significant improvement with our early warning sign project*,* we said – let’s change the policy to adapt to this change. The physician said*,* ‘Why will we change? Our old practice is good and functioning well.’ Even the chairperson of the paediatric department totally disagreed.”*

**FG2-C (FG2, doctoral group):** *“To change one letter in the policy*,* you have to wait three*,* four years for acceptance through committees. At the end*,* you will be tired of fighting all the hierarchy.”*

**FG2-D (FG2, doctoral group):** *“It is an obstacle in itself – a reluctance to change. For example*,* when I tell a senior colleague that these skills have not been updated*,* she says ‘No*,* that’s it – we have been used to this for years*,* so don’t try to lecture me.‘”*.

Translation: Translation facilitators were most evident when participants described organizational infrastructure that normalized EBP, mandatory training modules, workshops, and research support mechanisms (Table 4). Participants also described leadership-driven strategies strategic plans, annual goals, journal clubs and recognition mechanisms that encouraged implementation. Translation barriers were described in policy protocol change processes, including multi-level resistance from different professional groups and lengthy governance timelines where changes could take years (Table 4). The PET mapping (Table 5) indicates that participants articulated clear facilitators and barriers clustered around workload feasibility, hierarchical authority, evidence access, and slow system-level adoption pathways. In summary, the Translation domain reveals a paradox: the settings with the strongest facilitators (providing mandatory modules, and have strategic goals, as described by FG2 doctoral participants with leadership experience) also reported the most severe governance barriers (like multi-year approval timelines, and resistance). This co-existence suggests that infrastructure and governance constraints must be addressed for translation to succeed.

**Table 4:** Codebook PET domain: translation

Code	Definition	Include	Exclude
TRN-1 Organizational capacity-building	Structures supporting EBP implementation	Mandatory modules; workshops; research department	Individual motivation only
TRN-2 Leadership prioritization and goals	Strategic plans, unit goals, journal club expectations	Annual goals; journal clubs; recognition	Bedside workload resistance alone
TRN-3 Protocol integration examples	Translation of evidence into clinic	Adapted clinic approaches	Evidence definition only
TRN-4 Multi-level resistance	Pushback from clinicians/leadership during implementation	why will we change?	Database access issues
TRN-5 Governance delay	Slow committee approvals (years)	wait three, four years; change one letter	General culture without process

**Table 5:** PET MAPPING- Barriers and facilitators with organizational readiness indicators

PET domain	Theme	Barrier and facilitator	Description grounded in transcript	Indicators of strong readiness
Practice	EBP is not personal preference	Facilitator	EBP described as not favoritism, integrating research, experience, and patient prefrence.FG2-A: “Our practice is not based on favoritism… it is based on evidence.” FG2-B: “Practice relies on our experience and also on updated research, meaning what science has reached.”	Shared commitment to evidence guided decisions
Practice	Local adaptation of tools and protocols	Facilitator	Evidence review followed by implementation in local context; problem-solving orientation. FG2-C: “We adapted the early warning sign tool… we found a huge significant improvement in ambulatory admissions to the ICU.” FG1-B: “We revised the whole guideline on evidence-based practice” (Alteplase, Paediatric Oncology).	Local adaptation capacity demonstrated; contextual fit achieved. Units able to translate evidence into protocol change independently.
Practice	Workload resistance at bedside	Barrier	Resistance when EBP steps are perceived as added work. FG2-C: “The floor nurses were against it — why do we have to do all these things?” FG1-C: “I expect that the staff themselves are not always ready… it is difficult for them to let go of the previous one.”	High perceived task demands; low feasibility in workflow and reduced change efficacy.
Practice	Hierarchy limits nursing autonomy	Barrier	Follow-orders expectations FG2-D: “Nurses are expected to simply take instructions from the doctor, without initiative or independence in decision-making.” FG2-E: “When I tell a senior colleague skills have not been updated, she says ‘No, don’t try to lecture me.‘”	Nurse-led initiative not supported; role autonomy not formally recognized
Evidence	Variable EBP education and research literacy	Mixed	vary EBP preparation across both groups. FG2-D: “Many practitioners want to apply evidence, but unfortunately they lack basic research skills to determine if the evidence is appropriate.” FG2-G: “During my Master’s studies, there was a full course on evidence-based practice.” FG1-D: “Official training on EBP — I personally have never attended.”	Presence of formal training for some staff; awareness of evidence appraisal needs.
Evidence	Access to evidence resources/library; barriers when access is tied to accounts.	Barriers	Unstable access to journals to resource constraint lowering efficacy. Structural constraint: unstable access to journals and databases. FG2-H: “When my account is suspended or belongs to the university, I cannot search anymore — I cannot find free articles. It is tiring and exhausting.” FG1-E: “Resources are available from the hospital library, but access is inconsistent.”	Reliable institutional access to evidence databases not yet established for all staff. Digital library provision recommended.
Translation	Organizational EBP infrastructure	Facilitator	Mandatory training modules, workshops, and research support structures normalizing EBP. FG2-C: “We have a mandatory module for evidence-based practice — we have to do it every year. It is not optional.” FG1-F: “In KSUMC, there are courses conducted regularly for employees at various levels.”	Leadership engagement and training structures produce stronger change efficacy. Mandatory modules signal institutional commitment.
Translation	Leadership prioritization and goals	Facilitator	Strategic plans, annual goals, journal clubs, and recognition mechanisms encouraging implementation. FG2-C: “One of our strategic plans is research and EBP. Each unit sets its own annual goal and participates in a competition — nursing leaders are recognised for the most EBP projects.” FG1-G: “Encouragement is present but somewhat moderate — shortage reduces time available.”	Leadership-driven EBP goals and recognition systems active. Annual goals and journal club structures linked to individual performance plans
Translation	Slow, contested policy change	Barrier	Multi-level resistance FG2-C: “To change one letter in the policy, you have to wait three, four years for acceptance through committees. At the end, you will be tired of fighting all the hierarchy.” Also: physicians, chairpersons, and floor nurses each resisted the early warning sign policy change.	Weak readiness at governance level. Prolonged approval pathways reduce collective efficacy and motivation for EBP implementation.

Note: PET = Practice–Evidence–Translation model (Johns Hopkins). FG1 = Master’s-level students (*n* = 5); FG2 = Doctoral-level students (*n* = 5); *N* = 10. Quotations attributed by participant code

## Discussion

There is a coherent and convergent pattern that is noticed using PET guided analysis. Both groups the Master’s-level (FG1, *n* = 5) and doctoral-level (FG2, *n* = 5) groups described EBP in a similar way. They perceived it as a practical clinical decision-making method rather than an abstract research activity. In the same vein, they experienced barriers clustered around 1- workload feasibility, 2- hierarchical authority, and 3- evidence access. Otherwise, they identified translation facilitators centered around 1- organizational infrastructure and 2- leadership mechanisms (Table 5). Notably, all demonstrated convergence on all major themes. However, doctoral-level participants provided richer clinical implementation examples while Master’s-level participants see organizational processes and educational gaps as main themes. This help in supporting the credibility and saturation of the findings.

This PET mapping offers analytical value beyond simple categorisation: by separating bedside practice (Practice), evidence access and appraisal (Evidence), and organisational conversion of evidence into workflows (Translation), it reveals how barriers in one domain amplify constraints in others. For example, resistance in the Practice domain is compounded by the evidence access barriers of the Evidence domain. That is nurses who lack time to search literature are disadvantaged when database access is also unreliable. Similarly, Translation-level governance barriers neutralize the strong commitment of Practice-level EBP. This occurs when nurses are evidence-aware but institutionally blocked. This cross-domain interaction is a key analytical contribution that a barrier-facilitator list would not reveal. Our findings also reflect the institutional readiness framework proposed by Weiner [20], which identifies two core components: change commitment (shared motivation to pursue change) and change efficacy (collective confidence in executing it). The data reflects uneven readiness across the three PET domains and across institutional settings.

### Practice: EBP conceptualization and bedside barriers

The consistent framing of EBP as “not based on favoritism” and as an integration of evidence, experience, and patient preference (FG2-A; FG2-B) aligns with the Johns Hopkins PET model definition [3]. This shared conceptualisation across both educational levels reflects change commitment consistent with Weiner’s framework [20]. Both groups demonstrated local adaptation through clinical examples: a paediatric early warning score (FG2-C), a sedation assessment protocol achieving zero unplanned extubations over two years (FG2-C), and revision of an Alteplase dosing guideline in a paediatric oncology unit (FG1-B). This contextual adaptation is consistent with implementation science showing that local fit is a prerequisite for EBP adoption and sustainability [11]. Importantly, positive EBP beliefs alone do not guarantee implementation. As Stokke et al. [21] demonstrated that EBP beliefs and behaviour are associated but not equivalent. Additionally, they argued that structural conditions are required beyond motivation alone. The present findings reinforce this, participants across both groups expressed strong EBP values, yet that doesn’t mean translating into implementation due to practice-level barriers.

Both groups reported practice-level barriers. Workload resistance emerged when EBP steps were perceived as additional tasks: FG2-C noted floor nurses asked “why do we have to do all these things?”, and FG1-C observed staff find it “difficult to let go of the previous one.” This mirrors Saudi and regional findings [5, 13] consistently identifying time pressure and competing demands as primary barriers. Hierarchical norms constituted a further structural barrier: FG2-D described environments where nurses are expected to “simply take instructions from the doctor, without initiative or independence in decision-making,” and FG2-E described senior colleagues who refuse to update skills, saying “don’t try to lecture me.” These findings are consistent with Alqahtani et al. [5] and Cleary-Holdforth et al. [7] and with Middle Eastern and international literature on role boundaries limiting nurse-initiated change [9, 13, 22].

The hierarchical dynamic described is salient in the Saudi context, where cultural norms is formulated about seniority intersect with role boundaries in healthcare [7, 9]. This distinguishes the Saudi experience from Western contexts where nurse-initiated EBP change. In the western experiences, while still challenged, nurses might encounter less role-boundary constraints. Addressing this requires not only policy change, but a cultural shift in how nursing is valued within multidisciplinary teams.

In summary, workload resistance and limited autonomy indicate high perceived task demands and structural barriers that reduce change efficacy regardless of nurses’ commitment to change [20].

### Evidence: Research literacy and access constraints

There was many report in this study regarding variability in EBP education and research literacy. FG2-D noted that “many practitioners want to apply evidence, but unfortunately they lack basic research skills to determine if the evidence is appropriate,” while FG2-G described receiving a full EBP course during doctoral studies not before that and FG1-D reported never having attended a formal EBP workshop. This inconsistency is similar to the findings from Saudi nursing student studies showing that EBP beliefs and implementation competency are uneven across educational levels [3, 4]. Additional to that, broader literature indicates that appraisal confidence is an implementation bottleneck in clinicians [2, 6]. Moreover, The divergence between groups, doctoral participants more likely to have received formal EBP training. Due to that, a suggestion can be made that postgraduate education may serve as a partial moderator of research literacy gaps. Due to this, it could be argued that our findings can help with implications for curriculum design.

Equally important, another structural barrier emerged which is access to evidence resources. FG2-H described losing access to journals when her university account suspended, stating “tiring and exhausting,” while FG1-E noted that library access was inconsistent across hospital settings. Likewise, Alsadaan and Ramadan [6] and Alqahtani et al. [5], stated that lack of institutional resource as a key barrier to EBP uptake in Saudi settings. Indeed, from a readiness standpoint, change efficacy might be undermines due to unstable access to evidence constitutes [20].

### Translation: Leadership facilitation and governance barriers

Our strongest facilitators emerged in this study were in Translation domain. FG2-C described the hospital practice that supports the translation. A mandatory annual EBP modules, workshops, evidence-based projects required of all new graduates, and a nursing research department providing continuous education and funding. FG1-F reported similar institutional provision at their hospital. Leadership mechanisms were also prominent: FG2-C described many initiatives like with annual unit goals, journal clubs, and year-end competitions recognizing units completing the most EBP projects. These infrastructure and leadership processes represent the institutional signals that Weiner [20] identifies as strengthening change efficacy. In addition, it consistent with evidence showing that leadership behaviors, mentoring, and organizational support are vital to EBP [7, 10, 12, 23, 24].

Despite that, translation was limited due to slow and contested policy change processes. FG2-C provided a details about their attempting to embed an early warning score protocol into hospital policy, and the resistance they faced from floor nurses, physician resistance (“why will we change? Our old practice is good”), and chairperson-level opposition, before stating that “to change one letter in the policy, you have to wait three, four years for acceptance through committees.” This governance delay reflects a known pattern (bureaucracy) identified in Saudi and international literature in which motivated workers are blocked by prolonged governance timelines [1, 6]. Clearly, contested governance can be an indicator for weak readiness at the system level. Thus, collective efficacy decreases when staff expect implementation efforts will be stopped at any time regardless of motivation [20, 21].

### Cross-group convergence and implications

Undoubtedly, the convergence of themes between groups could be seen as a strengthen point that add credibility to the result. Both groups identified 1- workload resistance, 2- hierarchical constraints, and 3- variable evidence access as barriers. Likewise, they identified mandatory training structures and leadership mechanisms as main facilitators. However, the main difference was clinical experiences with EPB implementation. Significantly, doctoral participants described complex multi-stakeholder translation examples. In the other hand, Master’s participants frequently mentioned educational gaps and the need for curriculum integration.

This supports the argument for the need for direct implication on nursing education, leadership, and policy in Saudi Arabia. For nursing education, EBP competency (like literature searching, critical appraisal, and local adaptation) should be embedded as a core element in nursing curricula. Correspondingly, nursing leadership should protect EBP time by allocating specific concern to departmental EBP goals. It should be formalized as standard practice. Equally important, governance pathways for EBP protocol change are urgently needed. Nonetheless, structural barrier such as multi-committee, multi-year approval timelines documented in our data suggest that leadership commitment alone is not enough. To resolve this, a research committee, as suggested by FG2-E, with clear authority can helpful when evaluating EBP in a fast-track evidence-based protocol updates.

### Limitations

A number of limitations should be considered when interpreting these findings. First, the sample comprised *N* = 10 postgraduate nursing students enrolled at King Saud University (Master’s-level, FG1, *n* = 5; doctoral-level, FG2, *n* = 5), all within an academic setting and without frontline clinical nurses. These findings should not be generalised to frontline clinical nurses without postgraduate enrolment, or nurses in other Saudi institutions or regions. Participants’ perspectives, shaped by academic training, may differ from those of nurses working in clinical settings. Findings should therefore be interpreted as exploratory and contextually specific to this nursing population.

Second, although thematic convergence supports a degree of data saturation, the use of small groups (*n* = 5 each) might indicate that saturation cannot be confirmed. To address this, future research should use larger or multiple focus groups, or triangulating with individual interviews, to strengthen transferability.

Third, social desirability bias might be a struggle when forming focus group. This concern amplified by the hierarchical norms described by participants themselves. Whereby participants may have been less willing to express views that diverged from perceived norms or to openly criticize their institutions.

Fourth, the use of the Johns Hopkins PET framework deductively may have constrained the emergence of themes outside the three pre-defined domains. An inductive or hybrid approach might surface additional dimensions such as emotional labour, professional identity, or patient-level factors.

Fifth, interviews were online, which, while enabling participation across geographic locations, may have limited the depth of interaction.

Despite these limitations, this study makes a meaningful contribution by providing the first PET-framework-guided qualitative account of EBP barriers and facilitators in Saudi Arabia. Thereby, generating theoretically grounded complements the existing quantitative evidence in this context.

## Conclusion

Using the Johns Hopkins PET model, this study draws on data from two focus groups comprising Master’s-level (*n* = 5) and doctoral-level (*n* = 5) nursing students (*N* = 10) to show that nurses and nurse educators view EBP as practical decision making that integrates research, clinical experience, and patient preferences. Implementation is hindered when evidence-informed steps are perceived as added workload, hierarchical norms restrict nursing autonomy, research literacy and appraisal skills vary, and access to databases is unstable. Translation is strengthened by leadership prioritization, capacity-building, journal clubs, and research support, and slowed by contested governance and lengthy approvals.

## Data Availability

The datasets used and/or analyzed during the current study are available from the corresponding author on reasonable request.
